# Psychological pathways to responsible generative AI decision-making among teachers: governance clarity, perceived accountability, decision uncertainty, and AI literacy

**DOI:** 10.3389/fpsyg.2026.1989652

**Published:** 2026-10-28

**Authors:** Weiran Li, Yuhao Su

**Affiliations:** 1 Organization and Personnel Department, Shandong Communication & Media College, Jinan, Shandong, China; 2 Faculty of Education, Henan University, Kaifeng, Henan, China

**Keywords:** AI literacy, decision uncertainty, educational psychology, generative artificial intelligence, metacognitive monitoring, perceived accountability, teacher decision-making

## Abstract

**Introduction:**

Generative artificial intelligence (GenAI) can assist teachers with organizing information, generating alternatives, and drafting feedback. Its use in consequential judgments requires verifiable evidence and professional oversight. This study examines how teachers regulate optional GenAI assistance while preserving professional judgment.

**Methods:**

Research on felt accountability, metacognitive monitoring, and AI literacy is conceptually integrated to develop a psychological process model of teacher decision episodes. Governance clarity is specified through rule clarity and role clarity, with responsible GenAI decision-making defined as regulated decision conduct.

**Results:**

The conceptual analysis produces five testable propositions and one configurational expectation. Governance clarity is proposed to support responsible conduct through perceived accountability. Decision uncertainty is modeled as a metacognitive signal whose behavioral value depends on calibration and regulation; AI literacy is specified as a resource for verification, calibrated reliance, and appropriate escalation. Responsible conduct encompasses appropriate delegation, verification, calibrated reliance and accountable human authority, traceability, and escalation or override. These model outputs are theoretical propositions, not empirically established effects.

**Discussion:**

The framework distinguishes generative assistance from conventional algorithmic advice and identifies task-dependent applications in assessment, feedback, referral, and student support. Future tests should compare unaided and assisted judgment, distinguish process quality from decision accuracy, and examine stakes, reversibility, and contextual dependence. Teachers retain authority to accept, modify, or reject GenAI output, or proceed without GenAI assistance.

## 1 Introduction

Generative artificial intelligence (GenAI) offers teachers a conversational means of organizing supplied information, generating possible explanations, comparing options, and drafting feedback or communications. These affordances may be useful when teachers need to synthesize extensive material, consider an alternative interpretation, or formulate a response under time constraints. However, their availability does not establish that teachers need GenAI to make consequential judgments or that its output improves those judgments. Teachers already possess subject expertise, knowledge of students’ histories and classroom behavior, and relationships with learners and families that a model may not capture. The present framework starts from this professional knowledge and examines how optional GenAI assistance should be regulated when it enters a decision process.

The proposed added value is support for specific reasoning and communication activities, not an independent source of pedagogical authority. GenAI can help a teacher make existing evidence easier to inspect or generate an alternative worth considering; it cannot turn an unsupported interpretation into evidence about a particular student. Educational studies provide a bounded rationale for examining these uses. Research on ChatGPT feedback identifies both potential utility and quality limitations (Steiss et al., 2024), while a teacher-education experiment distinguishes improvements in diagnostic justification from improvements in decision accuracy (Kinder et al., 2025). These findings motivate investigation of selective assistance without establishing its necessity, superiority, or prevalence in high-stakes practice.

Teacher judgments about assessment, feedback, referral, student support, and family communication involve contextual knowledge, incomplete evidence, and unequal consequences. Human-automation research identifies risks of inappropriate reliance and rejection of advice (Parasuraman and Riley, 1997; Lee and See, 2004; Dietvorst et al., 2015; Logg et al., 2019). In an educational simulation, Henderson et al. (2026) found that educators generally favored their own judgments while sometimes consulting algorithmic advice. That study concerns algorithmic course recommendations; it is relevant background on selective reliance, rather than direct evidence of conversational GenAI use. The present question is how teachers evaluate and regulate generated material when it is considered for a consequential judgment.

International guidance increasingly places human agency, ethical judgment, transparency, and oversight at the center of educational AI. UNESCO’s guidance on GenAI emphasizes a human-centered approach, and its AI Competency Framework for Teachers identifies ethical understanding, critical evaluation, and professional capacity as central teacher competencies (Miao and Holmes, 2023; Miao and Cukurova, 2024). A systematic review of K–12 teachers’ ethical AI competencies similarly highlights human-in-the-loop reasoning, transparency and explainability, critical analysis, and ethical sensitivity (Tagare et al., 2025). Classroom-ready ethical guidance now translates abstract principles into role- and phase-specific teacher actions, while qualitative evidence suggests that teacher authority in the GenAI era is increasingly grounded in interpretive and ethical judgment rather than information control alone (Go et al., 2026; Zhang et al., 2026). These developments clarify what responsible practice should contain, but not the psychological pathways through which a teacher actually enacts it.

That translation is fundamentally psychological. Institutional rules do not act directly on a teacher’s choice; they must be noticed, interpreted, and represented as expectations about what can be done, what must be checked, and who remains responsible. A school may possess an extensive AI policy, yet a teacher can still be uncertain about whether it applies to a particular student-related task, whether an AI-generated recommendation requires independent verification, or when additional human review is warranted. Conversely, relatively concise guidance may be behaviorally powerful if the teacher clearly understands the decision boundary and anticipates being answerable for the process. The focal contextual construct is therefore governance clarity as experienced by the teacher, not policy existence in the abstract.

One psychological pathway is perceived accountability. Felt-accountability research defines accountability as the expectation that one’s conduct will need to be explained and justified to an audience capable of evaluation (Lerner and Tetlock, 1999; Hall et al., 2017; Schillemans et al., 2021). Such anticipated evaluation can change cognition before a decision is made by increasing attention to alternatives, anticipatory self-criticism, and information processing. Public-administration work likewise emphasizes that formal accountability arrangements matter partly through the felt answerability they produce in individual decision-makers (Overman and Schillemans, 2022). In AI-supported educational judgment, this mechanism is especially consequential because the system can generate the recommendation while the teacher retains professional responsibility for how that recommendation is evaluated, accepted, modified, rejected, recorded, or communicated.

A second psychological pathway concerns decision uncertainty and metacognitive monitoring. Decision uncertainty is the teacher’s subjective sense of how confidently the available evidence supports a judgment. Cognitive research shows that confidence and error monitoring are central to metacognition because people must estimate when their own decisions are reliable enough to act upon and when additional evidence is needed (Yeung and Summerfield, 2012; Fleming, 2024). GenAI can complicate this monitoring problem: fluency can create a feeling of evidential sufficiency even when the model is wrong, while opaque provenance can also create legitimate uncertainty about what should be trusted. Recent work on AI-assisted cognition therefore treats calibration—the fit between confidence, evidence, and actual reliability—as a central psychological challenge rather than assuming that more confidence or less uncertainty is always beneficial (Lee et al., 2025; Maier, 2026).

AI literacy may determine whether uncertainty becomes productive or disabling. Contemporary teacher-focused work increasingly defines AI literacy in terms of ethical use, critical evaluation, metacognitive monitoring, and behavioral regulation rather than mere familiarity with tools (Celik, 2023; Tagare et al., 2025; Kaya and Meylani, 2026; Gao and Wang, 2026). More literate teachers should be better able to recognize the limits of model output, distinguish confidence from evidence, protect student information, triangulate claims, and decide when a judgment should be escalated or kept human-only. In this framework, AI literacy is therefore a regulatory resource for calibrating reliance under uncertainty, not simply an antecedent of technology acceptance.

This article integrates these strands into a psychological process model of responsible GenAI decision-making among teachers. The model follows a context–cognition–action architecture rather than treating institutional policy as a direct cause of behavior. Governance clarity represents a teacher-perceived contextual cue; perceived accountability and decision uncertainty represent psychologically experienced states; and responsible GenAI decision-making (RADM) represents regulated decision conduct. AI literacy is treated as a person-level regulatory resource that changes how uncertainty is interpreted and converted into control. The model therefore connects institutional expectations to teacher cognition without reducing responsible AI either to policy compliance or to individual morality.

The explanatory focus is deliberately educational-psychological rather than administrative. Institutional governance is the context, but the theoretical problem concerns teachers’ anticipatory cognition, confidence and uncertainty, metacognitive monitoring, answerability, reliance calibration, and preservation of professional agency. This emphasis directly addresses current educational-psychology research on AI, which increasingly calls for psychologically grounded explanations of cognition, metacognition, beliefs, and self-regulation rather than surface-level accounts of technology use (Zhong and Simons, 2026). The article asks three linked questions: How does governance clarity make responsibility psychologically salient? How does recognized uncertainty alter monitoring and control of AI-supported evidence? Under what conditions does AI literacy help teachers convert uncertainty into calibrated, responsible action?

## 2 From AI adoption to responsible GenAI decision-making among teachers

### 2.1 GenAI-supported teacher judgment as a psychological decision context

The target phenomenon is the teacher decision episode: a situation in which a teacher considers or uses GenAI-generated material while forming a judgment that may affect a learner. GenAI is conceptualized primarily as a decision-support tool. Its appropriate functions include organizing information that the teacher is authorized to provide, drafting candidate feedback, generating alternative explanations, and suggesting questions for further inquiry. Substantive pedagogical judgment remains with the teacher and, where required, the relevant professional team. A generated interpretation is a candidate to evaluate, not new evidence about the student.

The framework distinguishes consultation from reliance. Consulting GenAI means viewing or eliciting output; reliance occurs when that output changes an interpretation, a communication, or a decision. A teacher may consult the system and reject its advice, or adopt a draft’s wording after independently establishing the underlying judgment. These behaviors should not be treated as equivalent. Reliance is warranted only to the extent that the contribution is relevant, independently checkable, and compatible with contextual evidence and institutional rules.

For example, a teacher reviewing several writing samples may ask an approved tool to organize rubric-relevant observations and draft alternative feedback statements. The teacher then checks each observation against the actual work and revises the feedback using knowledge of instruction, prior performance, and the learner’s needs. The possible benefit is reduced organization or drafting effort and a broader set of candidate responses. There may be little benefit when the evidence is already manageable, the output repeats what the teacher knows, or verification costs exceed any saving. A model’s suggestion that a learner has a particular need requires additional evidence; it does not justify a diagnosis or referral by itself.

Consultation is therefore most defensible when a permitted, bounded task can be specified, relevant evidence can be supplied without compromising confidentiality, and the teacher can evaluate the output. Teachers may proceed without GenAI when its contribution is unclear, sensitive context cannot be represented appropriately, or independent checking is infeasible. Appropriate non-use is part of responsible delegation. The model explains regulation within this range of choices; it does not predict universal adoption or assume that GenAI adds value in every decision.

The distinction between low-stakes assistance and consequential judgment is important. Brainstorming examples differs from drafting feedback that will be shared with a learner, assigning a recorded grade, or preparing material for a support or referral process. Consequences depend on how an output is used, not simply on the task label. Teachers must judge the permitted scope of assistance, the evidence required, and the need for additional professional review. Governance work supplies expectations for these decisions, while the present model concerns how those expectations are interpreted and enacted (Crompton et al., 2026; Go et al., 2026).

### 2.2 Defining responsible GenAI decision-making

Responsible GenAI decision-making (RADM) is defined here as a teacher’s selective, critical, transparent, and accountable use of GenAI input while preserving accountable human authority over the teacher’s contribution to a consequential educational judgment. RADM is intentionally a process construct rather than a synonym for decision accuracy. A final decision can be correct by chance even when the underlying process involves uncritical copying, hidden AI use, or inadequate checking; conversely, a carefully governed process may still produce a contestable outcome under genuine uncertainty. The construct therefore concerns how teachers regulate human–AI judgment, not whether AI is used at all.

Five behavioral dimensions are proposed. First, appropriate delegation concerns whether the teacher uses GenAI only for tasks and stages suitable for machine assistance. Second, verification refers to checking important claims, assumptions, and recommendations against independent evidence or professional knowledge. Third, calibrated reliance and accountable human authority require the teacher to weight AI output according to evidential quality and to modify, reject, override, or seek additional human judgment when stronger evidence warrants. Fourth, traceability involves documenting material AI involvement and the reasoning behind the teacher’s contribution to the final judgment when the stakes justify a record. Fifth, escalation and override involve pausing, seeking additional human review, or refusing AI-supported action when privacy, fairness, uncertainty, or potential harm exceeds an acceptable threshold.

These dimensions align with human-centered AI guidance, teacher ethical-competency research, and psychological work on appropriate reliance. They also make RADM potentially observable at the level of a decision episode. Rather than asking whether teachers endorse responsible-AI principles, researchers can examine whether they delegate appropriately, verify material claims, seek additional evidence, calibrate reliance, preserve accountable human authority, document consequential use, and escalate uncertainty in realistic scenarios. The five dimensions should initially be treated as non-interchangeable components of a higher-order behavioral composite, while future validation should compare composite/formative and correlated-factor alternatives rather than assuming a reflective scale structure in advance. This distinction is central to the present framework: responsible AI is treated as enacted regulation of judgment rather than as a favorable attitude toward ethics (see Table 1).

**Table 1:** Core constructs in the proposed model.

Construct	Working definition	Psychological function	Indicative operationalization
Governance clarity	Teacher-perceived clarity of rules, boundaries, procedures, review expectations, decision authority, and responsibility for GenAI-supported judgments.	Makes institutional expectations and answerability cognitively accessible; reduces avoidable procedural ambiguity.	Rule clarity; role clarity; allowed/prohibited uses; review and escalation expectations.
Perceived accountability	Expectation that one may need to explain and justify an AI-supported decision process to a legitimate and capable audience.	Promotes anticipatory self-criticism, information processing, and ownership of the reasoning process.	Expected scrutiny; answerability; review by a legitimate/competent forum.
Decision uncertainty	Subjective uncertainty about whether the available AI-supported evidence is sufficient and reliable for a consequential educational judgment.	Functions as a metacognitive monitoring signal; adaptive effects depend on calibration and available control strategies.	Evidential sufficiency; confidence; perceived missing context; need for additional evidence.
AI literacy	Knowledge, critical evaluation, ethical awareness, metacognitive monitoring, and behavioral regulation needed to use AI appropriately.	Supports calibrated reliance and connects uncertainty monitoring to verification, escalation, and override.	Knowledge of limits; critical evaluation; data/ethics awareness; monitoring; verification/override competence.
Responsible GenAI decision-making	Selective, critical, transparent, and accountable use of GenAI input while preserving accountable human authority in consequential educational judgment.	Behavioral regulation of human-AI judgment under institutional and evidential uncertainty.	Appropriate delegation; verification; calibrated reliance; accountable human authority; traceability; escalation/override.

### 2.3 Evidence on generative support and its distinction from algorithmic advice

Two strands of research inform the framework but serve different purposes. Studies of conventional algorithmic advice examine responses to predictions, scores, or recommendations and provide a basis for theorizing reliance, accountability, and override. Studies of GenAI in education address generated language and interaction around pedagogical tasks. Findings from the first strand are not sufficient evidence that teachers use, benefit from, or should rely on GenAI in consequential decisions.

Steiss et al. (2024) compared ChatGPT-generated formative feedback with feedback from trained human evaluators on secondary students’ writing. Human feedback was better on four of the five assessed quality dimensions. This supports examining generated feedback as a fallible draft requiring evaluation, rather than presuming that it replaces informed teacher feedback. The study assessed feedback quality; it did not validate automated grading or referral decisions.

Kinder et al. (2025) studied 269 German pre-service teachers completing diagnostic-reasoning tasks. Adaptive ChatGPT feedback improved the quality of written justification compared with static expert feedback, but did not significantly improve decision accuracy. This is direct evidence about generative support for pedagogical reasoning in a training setting. It does not establish effectiveness for in-service teachers making real decisions about students. Its distinction between justification and accuracy also cautions against treating more elaborate explanations as evidence of better judgments.

Compared with the fixed advice formats often examined in algorithmic-reliance studies, conversational GenAI permits iterative prompting, open-ended alternatives, and explanations whose wording changes with the interaction. The teacher helps construct the input and may repeatedly refine it, while plausible output can obscure unsupported assumptions. Consequently, monitoring includes checking what context was supplied, which assertions can be verified, whether rephrasing altered the underlying claim, and whether successive outputs merely repeat the same unsupported inference. Repeated agreement within a conversation is not independent corroboration. These interactional properties motivate extending accountability and metacognitive theory to GenAI, while leaving the extension open to empirical testing.

The psychological mechanisms proposed below are therefore not claimed to be unique to GenAI. Their application is shaped by the form of assistance, the interaction, and the educational task. The evidence reviewed supports investigating particular assistance functions; it does not demonstrate broad deployment in high-stakes teacher decisions or validate the proposed causal pathways.

### 2.4 Task differences and the limits of generative assistance

Accountability, uncertainty monitoring, and literacy may be relevant across tasks without operating with equal strength or producing identical behaviors. Table 2 distinguishes illustrative uses in assessment, feedback, referral, and student support. These are proposed applications and boundaries, not observed usage rates or validated prescriptions. Within each domain, the stakes, reversibility, verifiability, and dependence on contextual knowledge determine what assistance is appropriate.

**Table 2:** Illustrative task differences in GenAI decision support.

Decision context	Possible GenAI contribution	Human judgment and evidence	Regulatory emphasis
Assessment	Organize rubric-relevant observations or draft candidate comments.	Check the actual work, rubric, instructional context, and accommodations; retain grading authority.	Independent scoring checks and moderation when recorded outcomes matter.
Feedback	Draft alternative explanations or feedback wording.	Verify accuracy and select wording suited to the learner, prior instruction, and next steps.	Check tone and claims; revise before delivery; monitor student understanding.
Referral	Organize verified observations or prepare questions for a professional team.	Determine concern and referral grounds through authorized human procedures; GenAI cannot establish a diagnosis.	Privacy, provenance, documented evidence, and qualified human consultation.
Student support	Generate options or structure a support-plan draft.	Assess feasibility, learner preferences, prior responses, and contextual constraints.	Evaluate options, document material use, and review outcomes; escalate when needed.

The framework expects more checking, justification, and professional consultation as an output becomes harder to reverse or more consequential, but this does not imply greater reliance on GenAI. For formative feedback, regulation may involve checking a draft and inviting correction. For recorded assessment, it may require independent application of the rubric and moderation. For referral, it may mean using GenAI only to organize verified observations, followed by an authorized human process. For student support, it may involve comparing options with the learner’s circumstances and monitoring their effects. These distinctions concern the expression and possible strength of the proposed mechanisms; cross-task equivalence is an empirical question.

## 3 Theoretical foundations

### 3.1 A context–cognition–action architecture

The proposed model uses a context–cognition–action architecture to keep analytical levels distinct. Institutional arrangements do not directly determine a teacher’s behavior; they become psychologically relevant only insofar as teachers perceive decision boundaries, anticipate answerability, monitor the sufficiency of evidence, and select a control response. This architecture is deliberately parsimonious: the theoretical work is carried by felt-accountability research, metacognitive monitoring and confidence calibration, and AI-literacy research rather than by adding a broad technology-acceptance framework.

Governance clarity therefore functions as a contextual cue. Perceived accountability captures anticipatory answerability, while decision uncertainty captures the teacher’s monitored sense that available evidence may or may not justify action. RADM is the regulated behavioral response. AI literacy sits outside the main pathway as a person-level regulatory resource that can improve the translation of uncertainty monitoring into verification, reliance calibration, and appropriate escalation. The distinction between context, psychological state, and action is essential because the same written policy may produce different felt accountability, and the same level of subjective uncertainty may produce different behavior depending on the teacher’s regulatory resources.

### 3.2 Perceived accountability and anticipatory cognition

Accountability is relational. A decision-maker is accountable to an audience or forum that can demand an account and evaluate conduct. Psychological research focuses on felt or perceived accountability: the subjective expectation that one’s decisions and behavior will be evaluated and that justification may be required (Lerner and Tetlock, 1999; Hall et al., 2017). This expectation can alter cognition prospectively. When people anticipate having to justify a choice to a legitimate audience whose preferences are not fully known, they are more likely to consider alternatives, engage in anticipatory self-criticism, and process decision-relevant information more systematically.

This mechanism is especially relevant to GenAI-assisted teacher judgment because algorithmic input can blur experienced agency. The system may generate an explanation or recommendation, but the teacher still decides how it is evaluated, weighted, modified, rejected, recorded, or communicated within the teacher’s role. If institutional arrangements leave responsibility diffuse—‘the model suggested it’ or ‘the policy did not say who checks it’—answerability can become psychologically attenuated. By contrast, explicit expectations about review and accountable human authority can make the teacher’s professional responsibility salient before the decision is finalized. Evidence from public-accountability research supports this micro-foundational logic: de facto accountability practices shape the felt expectation of having to explain and justify decisions (Schillemans et al., 2021; Overman and Schillemans, 2022).

Accountability is not uniformly beneficial. Outcome-only or punitive accountability can encourage defensiveness, impression management, or risk avoidance. For responsible GenAI use, the theoretically relevant form is process accountability: the expectation that a teacher can explain what evidence was used, how AI output was checked, why the final judgment was made, and how uncertainty was handled. The model therefore links governance clarity to accountability through cognitively usable answerability, not fear of sanction.

### 3.3 Decision uncertainty as a metacognitive signal

Decision uncertainty arises when a teacher lacks confidence that the available evidence, criteria, or options support a sufficiently justified choice. From a metacognitive perspective, uncertainty is not merely missing information; it is information about one’s own decision state. Confidence judgments and error monitoring help people estimate whether a decision is reliable enough to act upon or whether additional processing is warranted (Yeung and Summerfield, 2012; Fleming, 2024). In educational psychology, monitoring accuracy is consequential because regulation depends on noticing when one’s current understanding or judgment may be inadequate, and monitoring can be improved through targeted strategy instruction (Gutierrez de Blume, 2022).

GenAI complicates this monitoring process because it can simultaneously reduce task effort and distort confidence cues. A coherent answer can increase subjective certainty without increasing evidential quality, while opaque provenance may produce legitimate uncertainty even when an output is useful. Human–automation research therefore emphasizes appropriate reliance rather than maximal trust (Lee and See, 2004). Recent work makes the metacognitive connection explicit: confidence and trust can become miscalibrated when reliability cues and actual accuracy dissociate, and metacognitive sensitivity has been proposed as a key mechanism for deciding when AI advice should be incorporated (Lee et al., 2025). Conceptual work on AI-assisted cognition similarly warns that improved task performance can coexist with poorer self-evaluation when AI promotes cognitive substitution or erroneous attribution of success (Maier, 2026).

For teachers, the critical issue is therefore not to eliminate uncertainty but to diagnose and regulate it. Subjective uncertainty may arise from at least two sources: uncertainty inherent in the learner case and evidence, and avoidable uncertainty about how AI output should be handled within the institutional process. Clear governance can reduce the latter, but it cannot remove genuine epistemic uncertainty in the former. More importantly, low subjective uncertainty can be maladaptive when it reflects fluency-based overconfidence, whereas recognized uncertainty can function as a metacognitive cue that motivates checking, comparison, and consultation. Very high uncertainty can also be difficult to regulate when the teacher lacks a strategy for identifying what to verify or which source to trust. The theoretical emphasis is therefore calibration—whether the teacher’s confidence and uncertainty are proportionate to the evidential situation and connected to an appropriate control response.

### 3.4 AI literacy as a metacognitive and ethical regulatory resource

AI literacy was initially discussed largely in terms of understanding what AI is and how to use it, but recent educational frameworks emphasize evaluation, ethics, monitoring, autonomy, and human agency. UNESCO’s teacher framework combines a human-centered mindset, ethics of AI, AI foundations and applications, AI pedagogy, and professional learning (Miao and Cukurova, 2024). Empirical work has extended teacher knowledge frameworks to include ethical assessment of AI-based decisions (Celik, 2023), while recent teacher-focused instruments distinguish awareness, use, evaluation, ethics, metacognitive monitoring, behavioral regulation, and critical evaluation as meaningful components of AI literacy (Kaya and Meylani, 2026; Gao and Wang, 2026).

This broader conception makes AI literacy especially relevant to decision uncertainty. More literate teachers should be better able to distinguish uncertainty in the educational case from uncertainty introduced by the model, recognize hallucination or bias risks, compare AI output with independent evidence, protect sensitive data, and decide when a task exceeds the appropriate boundary for AI assistance. Literacy therefore does not simply increase confidence; it should improve the teacher’s capacity to interpret uncertainty and select an appropriate control response. In metacognitive terms, AI literacy supplies strategies that connect monitoring to action.

## 4 Proposed model and propositions

Figure 1 presents the proposed psychological process model for episodes in which teachers consider or use GenAI as decision support. The model is conditional on task suitability and on a potential contribution that can be checked against evidence and professional knowledge. Governance clarity is a contextual cue; the explanatory core concerns accountability, uncertainty monitoring, calibration, and regulation of reliance. The five propositions concern this regulatory process, not the necessity or effectiveness of GenAI adoption. Task characteristics discussed in Sections 2.4 and 5 may change the strength and behavioral expression of the pathways.

**Figure 1:**
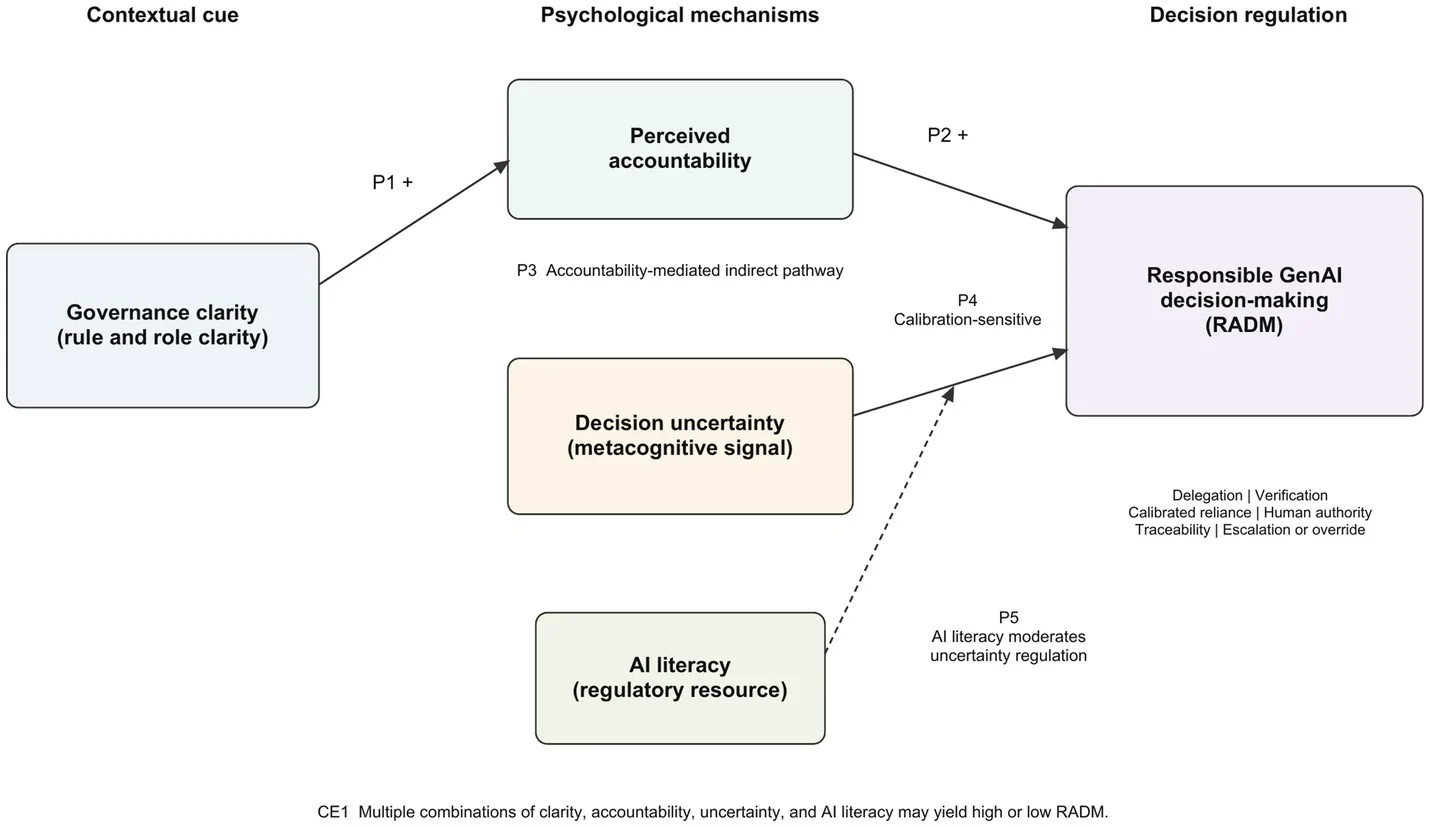
Proposed psychological process model of responsible GenAI decision-making among teachers. The pathways concern regulation of optional assistance; they do not imply that GenAI improves every decision. Task suitability, stakes, reversibility, and contextual dependence bound the model. P1-P5 are testable propositions; CE1 is a secondary configurational expectation.

### 4.1 Governance clarity as a contextual cue

Governance clarity refers to the extent to which teachers perceive institutional expectations for GenAI-supported judgments as understandable, operational, and role-specific. It is proposed as a higher-order contextual construct with two related facets whose empirical distinctiveness should be tested rather than assumed. Rule clarity concerns what uses are permitted, restricted, or prohibited; what evidence or disclosure is required; what student information may be entered; and when independent human checking or escalation is mandatory. Role clarity concerns who is authorized to use GenAI, who must verify material output, who retains accountable human authority for the teacher’s part of the decision process, and who should be consulted when competence or certainty is insufficient.

This distinction builds on organizational research showing that clarity is a distinct quality of information and that role ambiguity arises when individuals do not know what is expected of them or how much authority they possess (Rizzo et al., 1970; Schnackenberg and Tomlinson, 2016; Schnackenberg et al., 2021). For teachers, rules without roles can leave responsibility diffuse, whereas roles without usable rules can create answerability without sufficient procedural guidance. Governance clarity therefore matters psychologically when it reduces interpretive work about the institutional process while preserving the teacher’s need to evaluate the educational evidence itself.

Clear rules and roles should increase perceived accountability because they identify both the decision boundary and the locus of answerability. When teachers know that material AI output must be checked and that they remain answerable for how they use it, responsibility is harder to transfer psychologically to the system. Governance clarity may also reduce avoidable procedural uncertainty about what constitutes an acceptable review process, but the model does not predict that it necessarily lowers substantive uncertainty about the learner case or makes AI output more reliable. For this reason, no direct proposition is advanced from governance clarity to the level of decision uncertainty.

*Proposition 1 (P1)*. Greater governance clarity will be associated with higher perceived accountability for GenAI-supported teacher judgments.

The predicted effect is strongest when rules and roles are not merely available but behaviorally usable: teachers can retrieve them at the point of decision, understand the evidence or review threshold they imply, and identify a legitimate audience to whom the process could be explained.

### 4.2 Perceived accountability and responsible GenAI decision-making

Perceived accountability should encourage RADM because anticipated justification changes the teacher’s processing goal. A teacher who expects to explain a consequential judgment has stronger reason to inspect the evidence, notice unsupported assumptions, consider alternative interpretations, and preserve a record of how the decision was reached. In GenAI-supported judgment, these behaviors correspond closely to verification, calibrated reliance, traceability, and explicit human responsibility for the reasoning process.

The strongest prediction applies when accountability is process-oriented and the evaluating audience is perceived as legitimate and competent. If teachers believe that colleagues, school leaders, families, or review bodies can ask how GenAI information was used and can meaningfully evaluate that explanation, accountability should be cognitively consequential. If the audience is perceived as symbolic, punitive, or interested only in outcomes, the effect may be weaker or defensive. The core proposition therefore concerns expected answerability for the quality of the reasoning process.

*Proposition 2 (P2)*. Higher perceived accountability will be associated with more responsible GenAI decision-making, expressed through greater verification, calibrated reliance, accountable human authority, traceability, and appropriate escalation or override.

*Proposition 3 (P3)*. Perceived accountability will mediate a positive relationship between governance clarity and responsible GenAI decision-making.

### 4.3 Decision uncertainty, calibration, and monitoring

A simple linear assumption that less uncertainty is always better is unlikely to capture responsible human–AI judgment. Metacognitive research distinguishes confidence from accuracy: feeling certain does not guarantee being correct, and adaptive regulation depends on whether confidence is calibrated to the evidence (Fleming, 2024). In GenAI contexts, fluent language can become a misleading cue to certainty. When teachers experience an AI-supported interpretation as self-evidently correct, they may see little reason to check sources, compare alternatives, or seek contextual information.

Recognized uncertainty can become a useful monitoring cue when it is proportionate to the evidential situation and connected to a feasible control strategy. A teacher who notices that a recommendation may be incomplete, contestable, or sensitive to missing context can respond by searching for information, checking records, consulting another professional, or delaying action. Research on metacognitive monitoring suggests that monitoring is useful because it informs control decisions—what to inspect, whether to continue processing, and when to seek additional evidence (Yeung and Summerfield, 2012; Gutierrez de Blume, 2022). At the other extreme, uncertainty that exceeds the teacher’s capacity to diagnose or resolve it may produce indecision, undifferentiated distrust, or deference to AI as an external authority.

The resulting prediction is calibration-sensitive rather than a strong claim that one universal amount of uncertainty is optimal. Future tests should measure objective ambiguity or evidential quality alongside subjective confidence so that warranted certainty can be distinguished from overconfidence and productive uncertainty from poorly regulated indecision. The most theoretically diagnostic outcome is not uncertainty alone, but whether teachers recognize when additional control is needed and act accordingly.

*Proposition 4 (P4)*. The relationship between subjective decision uncertainty and responsible GenAI decision-making will be calibration-sensitive: recognized and regulable uncertainty will be associated with more verification, information search, and appropriate escalation than unwarranted certainty or uncertainty that exceeds the teacher’s available control strategies.

### 4.4 AI literacy as a boundary condition on uncertainty regulation

AI literacy should alter the behavioral meaning of uncertainty. Low-literacy teachers may be unable to diagnose why an AI output is uncertain, distinguish plausibility from evidential support, or identify a suitable verification strategy. More literate teachers have a richer regulatory repertoire: they can compare outputs with independent evidence, identify data or bias risks, recognize when additional prompting does not validate an answer, and know when professional or specialist review is required.

AI literacy should also protect against misplaced certainty. More literate teachers are better positioned to understand that linguistic fluency is not equivalent to accuracy and that GenAI systems can produce confident errors. Recent AI-literacy work places critical evaluation and metacognitive monitoring at the center of competent use, while work on AI-assisted cognition warns that performance support can produce miscalibrated self-evaluation if users stop monitoring (Kaya and Meylani, 2026; Maier, 2026). AI literacy should therefore improve the translation of uncertainty monitoring into appropriate control rather than simply raise or lower confidence.

*Proposition 5 (P5)*. AI literacy will moderate uncertainty regulation such that, at higher levels of AI literacy, recognized decision uncertainty will be more strongly associated with verification and appropriate escalation, and unwarranted confidence will be less likely to produce uncritical reliance on GenAI output.

### 4.5 A configurational extension: multiple psychological pathways to responsibility

The preceding propositions describe directional psychological pathways, but responsible GenAI decision-making may also be conjunctural. One teacher may act responsibly because clear institutional expectations create strong accountability; another may compensate for incomplete local guidance through high AI literacy and disciplined verification; a third may face substantial uncertainty but respond responsibly because both accountability and regulatory competence are high. Conversely, low responsible conduct may arise through different combinations, such as low clarity plus low accountability or high uncertainty plus low literacy even when formal rules exist.

This equifinality matters because educational interventions should not assume one universally necessary condition. Individual literacy cannot indefinitely substitute for clear institutional responsibility in consequential decisions, and clear policy cannot guarantee responsible behavior if teachers cannot evaluate AI output. A configurational perspective therefore complements the directional process model and is appropriate for heterogeneous educational settings in which institutional support and teacher competence vary together.

#### 4.5.1 Configurational expectation (CE1)

High and low responsible GenAI decision-making may exhibit configurational asymmetry: multiple combinations of governance clarity, perceived accountability, decision uncertainty, and AI literacy can be associated with similarly high or low RADM (see Table 3).

**Table 3:** Propositions and recommended empirical tests.

Proposition or expectation	Core relationship	Primary test	Key caution
P1	Governance clarity → perceived accountability (+)	SEM / multilevel SEM	Treat clarity as teacher-perceived and task-specific; test rule/role facets.
P2	Perceived accountability → RADM (+)	SEM / scenario experiment	Emphasize process answerability rather than punitive/outcome-only accountability.
P3	Governance clarity → accountability → RADM	Bootstrapped indirect effect / temporally separated mediation	Do not infer causality from a single cross-sectional survey.
P4	Decision uncertainty → RADM (calibration-sensitive)	Scenario experiment; polynomial or calibration-sensitive models	Measure objective ambiguity/evidence quality alongside subjective confidence.
P5	AI literacy × uncertainty regulation → RADM	Moderated regression / SEM / scenario interaction	Model critical evaluation and metacognitive regulation, not only tool proficiency.
CE1	Equifinal configurations → high/low RADM	fsQCA after construct validation	Treat as a secondary configurational extension; report calibration and robustness transparently.

## 5 Boundary conditions and rival explanations

### 5.1 Educational stakes and reversibility

Educational stakes and reversibility should influence the strength and expression of the model’s pathways. A draft feedback comment that can be revised before delivery differs from a recorded grade or material entering a referral process. Task categories alone do not determine risk: formative feedback can still mischaracterize a learner, and administrative preparation for a referral can remain separate from the referral judgment itself. Higher stakes and lower reversibility are expected to increase the need for verified evidence, process accountability, and appropriate consultation, sometimes making non-use the responsible choice. The same level of checking and documentation should not be assumed appropriate for every task. These are boundary expectations requiring cross-task tests, not established moderator effects.

### 5.2 AI capability, transparency, and domain fit

Uncertainty is partly produced by the technology. Systems that provide sources, calibrated confidence cues, or domain-relevant evidence may support better monitoring than systems that generate unsupported prose. Yet explanation alone should not be assumed to solve the problem of reliance. Human–AI research shows that transparency or confidence cues can increase trust without necessarily improving accuracy, making verifiability and calibration more important than explanation as an end in itself (Lee et al., 2025). Technical design is therefore a boundary condition on the teacher’s psychological task, not a replacement for professional judgment.

### 5.3 Organizational climate, leadership, and psychological safety

Governance clarity operates within a broader climate. Leaders who reward speed while formally requiring careful review create contradictory cues. Psychological safety also matters: teachers are more likely to acknowledge uncertainty, seek a second opinion, or escalate an AI-supported judgment when doing so is not treated as evidence of incompetence. Future studies should therefore distinguish written rule clarity from an enacted climate that makes reflective monitoring and admission of uncertainty socially possible.

### 5.4 Teacher expertise and task familiarity

Teachers differ in subject expertise, assessment experience, knowledge of individual students, and familiarity with particular tasks. These resources establish the baseline against which GenAI’s incremental contribution must be assessed. Rich contextual knowledge can make generated suggestions redundant or reveal why an apparently plausible explanation is inappropriate. Conversely, an unfamiliar task or extensive material may make assistance attractive while also increasing verification demands. Henderson et al. (2026), studying conventional algorithmic advice, provide background evidence of selective rather than uniform educator reliance; this should not be generalized directly to GenAI. Future work should test teacher expertise, student familiarity, task complexity, and prior GenAI experience as possible influences on both consultation and regulation.

### 5.5 Cultural and institutional context

Accountability norms differ across education systems and professional cultures. In some contexts teachers anticipate hierarchical review; in others, collegial moderation, family scrutiny, professional discretion, or legal due process may be more salient. The framework predicts psychological mechanisms rather than one universal governance design. Cross-cultural work should therefore examine whether the same constructs operate through different accountability audiences and whether rule and role clarity carry the same psychological meaning across systems.

## 6 Methodological agenda for empirical testing

The model is intentionally testable and is designed for a staged research program rather than a single cross-sectional survey. The empirical priority should be to validate the psychological constructs and then separate contextual perception, internal states, and decision behavior over time. A strong program would combine measurement development, temporally separated survey evidence, controlled educational decision scenarios, behavioral indicators, and—after the main constructs are established— configurational analysis.

Study 1 should validate the measurement model with in-service teachers who have meaningful experience using GenAI. Governance clarity can be operationalized with separate rule-clarity and role-clarity indicators, adapting organizational clarity measures to concrete educational decisions (Rizzo et al., 1970; Schnackenberg et al., 2021). Perceived accountability should emphasize expected answerability and evaluation rather than conscientiousness or generic professional responsibility (Hall et al., 2017; Overman and Schillemans, 2022). Decision uncertainty should be assessed as subjective confidence in evidential sufficiency and, where possible, paired with objective ambiguity or evidence-quality indicators so that calibration can be evaluated. AI literacy should emphasize critical evaluation, ethical judgment, monitoring, and regulation rather than frequency of AI use (Celik, 2023; Tagare et al., 2025; Kaya and Meylani, 2026; Gao and Wang, 2026).

RADM requires the greatest measurement care because it is a behavioral-process construct. The five proposed dimensions are non-interchangeable: a teacher can verify an output without documenting it, or can protect privacy without calibrating reliance. Initial instrument development should therefore compare a higher-order composite/formative specification with correlated first-order behavioral dimensions rather than assuming a reflective factor. Validation should use scenario-based choices and process indicators, not self-report endorsement alone. For example, teachers can be presented with an AI-generated interpretation of a student-support or assessment case and asked what they would do next. Choices can capture delegation, independent verification, information search, data protection, reliance weighting, documentation, consultation, and willingness to override the recommendation. Convergent validity should be demonstrated with critical AI evaluation and ethical competence; discriminant validity should be established against general AI acceptance, trust, self-efficacy, and social desirability.

Study 2 should use a temporally separated design. At Time 1, teachers can report governance clarity, AI literacy, role, prior AI experience, and contextual controls. At Time 2, perceived accountability can be measured with reference to the teacher’s most recent substantive GenAI-supported judgment, together with subjective uncertainty and perceived evidential sufficiency. At Time 3, RADM can be assessed through retrospective process items and a standardized scenario battery. A three-wave design reduces same-time common-method inflation and better approximates the proposed context–cognition–action sequence. If teachers are sampled within schools, multilevel modeling can test whether shared school-level clarity explains variance beyond individual perceptions; aggregation should be justified empirically rather than assumed from individual reports.

Study 3 should use a staged experimental program rather than combine all task and system features into one underpowered design. An initial study can compare unaided teacher judgment, judgment with a conventional fixed recommendation, and judgment with conversational GenAI assistance on matched cases. Each condition should provide the same underlying student evidence; AI-output quality and available task information should be controlled or recorded. Teachers should make an initial judgment and confidence rating before assistance, where applicable, and a final judgment afterward. This distinguishes consultation, acceptance of wording, and substantive reliance, and tests whether assistance adds value beyond professional judgment.

A follow-up experiment can manipulate governance clarity through explicit permitted uses, review requirements, decision authority, and escalation rules, while varying evidential completeness or output reliability. Task batteries should include assessment, feedback, referral, and student-support scenarios with independently specified stakes and reversibility. Multilevel analyses can separate teacher and episode variation; measurement invariance or task-specific indicators should be examined before comparing pathway estimates. Prespecified interactions can test whether stakes, verifiability, or contextual dependence change the accountability and uncertainty-regulation pathways. Not every scenario needs to permit AI use: justified non-use or referral to a qualified professional should be available responses.

Outcomes should distinguish process quality, task performance, and practical cost. Verification, information search, documentation, and escalation measure regulation; expert-rated judgment quality or accuracy, where a defensible criterion exists, assess outcomes; time and workload assess efficiency. For contestable cases, blinded expert evaluation of evidence use and justification is preferable to inventing a single correct answer. Confidence should be compared with these criteria rather than with the fluency or length of the response. An increase in checking alone does not establish better decisions or a net benefit from GenAI assistance.

Study 4 can use fsQCA as a secondary extension after the main constructs are validated. Governance clarity, perceived accountability, AI literacy, and calibrated or scenario-specific uncertainty can be treated as conditions and RADM as the outcome. The purpose is not to convert a methodological preference into a theory claim or to duplicate SEM, but to examine whether distinct combinations of institutional clarity and psychological resources are associated with similarly responsible behavior. Calibration anchors, necessary-condition tests, truth-table thresholds, consistency, coverage, contradictory configurations, and robustness checks should be fully reported.

Across studies, researchers should avoid treating self-reported intention as equivalent to responsible behavior. Scenario choices, process-trace measures, confidence ratings, information-search behavior, decision logs, or peer/supervisor assessments are preferable when feasible. The unit of analysis should be the GenAI-supported teacher decision episode: what the model contributed, what the teacher checked, how confident the teacher was, what uncertainty remained, what additional evidence was sought, and where accountable human authority resided in the decision process.

## 7 Discussion

### 7.1 Theoretical contributions

The framework makes four contributions. First, it shifts the outcome of educational AI research from adoption or intention to a behavioral process of responsible judgment. Technology-acceptance models can explain whether teachers are willing to use AI, but they do not specify how teachers decide what to delegate, what to verify, when to override, what to document, or when to seek another human judgment. RADM therefore turns principle-level responsible-AI language into a decision-regulation problem aligned with educational psychology’s concern for cognition and self-regulation.

Second, the model provides a psychological link between institutional AI expectations and teacher behavior. Governance research identifies transparency, human oversight, literacy, and accountability, but institutional arrangements influence behavior only after teachers perceive and interpret them. Perceived accountability specifies how clear rules and roles can become anticipatory answerability. The model deliberately avoids assuming that policy clarity removes substantive uncertainty in the learner case; instead, it treats uncertainty as a separate metacognitive state that teachers must monitor and regulate.

Third, the framework connects decision uncertainty with metacognitive calibration. Educational psychology has long treated monitoring as a prerequisite for regulation, yet responsible-AI discussions often treat uncertainty mainly as a technical defect to be reduced. The present framework instead proposes that recognized uncertainty can be behaviorally useful when it triggers checking and consultation, while unwarranted certainty and unregulated uncertainty can undermine responsible action. AI literacy is positioned as a regulatory resource that helps translate monitoring into control.

Fourth, the configurational extension recognizes that teachers operate in heterogeneous institutional environments and may reach responsible practice through different combinations of contextual clarity, accountability, uncertainty, and AI literacy. This extension is subordinate to the psychological process model: it is intended to test equifinality after the core constructs and directional propositions are validated, not to substitute set-theoretic results for mechanism evidence.

Taken together, these contributions explain responsible GenAI use as regulated professional judgment rather than as compliance, trust, or technical competence alone. The model is intentionally bounded: it does not claim that governance clarity is sufficient, that uncertainty should always be increased, or that AI literacy can compensate indefinitely for weak institutional responsibility.

### 7.2 Implications for teacher practice and institutional design

The practical implication is not that schools need longer AI policies. They need psychologically usable governance: a small number of consequential boundaries that teachers can retrieve and apply while making decisions. Guidance should make clear which tasks may receive GenAI assistance, what student information may not be entered, which outputs require independent checking, where accountable human authority resides, and when additional review is required. Examples and counterexamples are likely to support cognition more effectively than broad exhortations to use AI ‘responsibly.’

Role clarity is equally important. Every consequential GenAI-supported workflow should answer four questions for the teacher: What am I allowed to delegate? What am I responsible for verifying? Where does accountable human authority reside? Who should I consult when uncertainty or risk exceeds my expertise? These questions convert abstract accountability into a decision architecture and make it less likely that responsibility diffuses across a teacher, school leader, vendor, and system.

Professional development should focus on uncertainty calibration and metacognitive monitoring rather than prompt engineering or productivity alone. Teachers need practice distinguishing fluency from evidential strength, recognizing missing context, comparing AI output with authoritative sources, protecting sensitive student information, and deciding when to seek a second opinion. Scenario-based professional learning can make these skills concrete by asking teachers to state not only what decision they would make, but how confident they are, what they would verify, and why they accepted, modified, rejected, or escalated the AI-supported recommendation.

Finally, documentation should be proportional to educational stakes. Routine low-risk use does not require bureaucratic logging of every interaction. Consequential uses should preserve enough process evidence to reconstruct the teacher’s reasoning: the GenAI contribution, independent evidence checked, uncertainty identified, and basis for the final action. Such traceability supports reflective practice and accountability without turning responsible AI into paperwork for its own sake.

### 7.3 Limitations and future research

The psychological process model developed in this study has not yet been empirically validated. Its proposed relationships should therefore be interpreted as testable propositions rather than established causal effects. The calibration-sensitive prediction for decision uncertainty is especially provisional. It should be evaluated against objective task ambiguity, evidence quality, and decision accuracy so that warranted certainty can be distinguished from overconfidence and productive uncertainty from poorly regulated indecision.

Direct evidence on GenAI in real, consequential teacher decisions remains limited in the literature examined here. Feedback-quality studies and pre-service teacher experiments inform possible assistance functions but do not establish effectiveness in referral, placement, or sustained student-support practice. The task examples in Table 2 and the cross-task expectations are theoretical elaborations. Future field studies should document what teachers actually consult, how they use or reject generated material, and whether any benefit exceeds verification costs and risks. Such studies must allow for the possibility that unaided professional judgment is preferable in some settings.

The constructs also require careful discrimination. Governance clarity is narrower than organizational transparency and should be task-specific; its rule- and role-clarity facets may prove empirically separable. Perceived accountability concerns anticipated answerability to a legitimate evaluating audience rather than generic conscientiousness or professional duty. AI literacy overlaps with self-efficacy and ethical competence but should not be reduced to confidence in using tools. RADM is intentionally process-focused and its higher-order structure is provisional, requiring comparison of composite/formative and correlated-factor specifications as well as validation against behavioral and calibration criteria.

Future research should examine objective and subjective uncertainty separately. A teacher can feel certain in a genuinely ambiguous case or uncertain in a well-specified one. The gap between evidential uncertainty and subjective confidence may itself be a key indicator of metacognitive calibration. Experimental designs that vary information quality, model reliability, and uncertainty cues can test whether governance clarity and AI literacy improve regulation and calibration rather than merely increasing confidence (Fleming, 2024; Lee et al., 2025).

Finally, the model is designed primarily for in-service teacher judgments in which GenAI can influence learners’ assessment, support, or educational treatment. It may generalize to higher education, advising, learning analytics, or other professional educational decisions, but such generalization should be tested rather than assumed. Accountability audiences, professional discretion, data sensitivity, and the meaning of uncertainty differ across systems and cultures.

## 8 Conclusion

When teachers consider GenAI for educational decisions, its contribution must be established at the level of a task and a decision episode. Organizing information, generating alternatives, or drafting feedback can offer support, but generated language does not replace evidence about a learner or the teacher’s contextual and professional knowledge. The educational-psychological question is how teachers monitor uncertainty, experience answerability, and regulate this optional assistance when it could shape a consequential judgment.

The framework developed here explains that translation through governance clarity, perceived accountability, decision uncertainty, and AI literacy. Clear rules and roles make answerability cognitively accessible without eliminating genuine uncertainty in the learner case. Perceived accountability encourages teachers to own and justify the decision process. Decision uncertainty functions as a metacognitive signal whose value depends on calibration and available control strategies. AI literacy helps teachers interpret that signal, verify evidence, and decide when to accept, modify, reject, or escalate an AI-supported recommendation. Responsible GenAI decision-making is therefore neither simple compliance nor maximal skepticism; it is the regulated preservation of accountable human professional agency.

This framework reframes responsible AI in education as a problem of teacher decision psychology embedded in an institutional context. It offers testable pathways from contextual clarity to anticipatory accountability, from uncertainty monitoring to calibrated reliance, and from AI literacy to responsible control. In doing so, it provides a basis for research that asks not only whether teachers use GenAI, but how they remain answerable for the judgments they make with it.
