# Supplementary material for: Psychological pathways to responsible generative AI decision-making among teachers: governance clarity, perceived accountability, decision uncertainty, and AI literacy
Source: Front Psychol. 2026 Oct 28;17:1989652. doi: 10.3389/fpsyg.2026.1989652 (PMC13751961; doi:10.3389/fpsyg.2026.1989652)
Supplement: Supplementary file 1 [file Table_1.docx]

# Supplementary Material S1. Proposed measurement items for future empirical validation

Associated manuscript: Psychological pathways to responsible generative AI decision-making among teachers: governance clarity, perceived accountability, decision uncertainty, and AI literacy

Author: Yuhao Su | Faculty of Education, Henan University, Kaifeng, Henan, China | ORCID iD: 0009-0008-5687-941X

The items below are illustrative and are intended to operationalize the proposed constructs in future research. They are not a validated scale. Before substantive use, they require expert content review, cognitive interviewing where feasible, pilot testing, assessment of dimensionality, reliability, convergent and discriminant validity, and validation against behavioral or scenario-based criteria.

## **Governance clarity – rule clarity (illustrative)**

The school’s expectations about when GenAI may be used in formal student-related decisions are clear.

I understand which GenAI-supported tasks require independent human verification.

The school provides clear guidance about what student information may or may not be entered into GenAI systems.

The conditions under which an AI-supported judgment must be escalated for additional review are clear.

## **Governance clarity – role clarity (illustrative)**

I know where accountable human authority resides when GenAI contributes to a student-related judgment.

I know what I am personally responsible for checking when I use GenAI in a consequential educational decision.

I know who should review an AI-supported judgment when the case is uncertain or high risk.

I clearly understand my responsibility for explaining how AI-generated information influenced my part of the decision process.

## **Perceived accountability (illustrative adaptation targets)**

I expect that I may need to explain how I used AI-generated information in a consequential educational decision.

I expect that the reasoning behind my AI-supported decisions can be reviewed by others.

I feel answerable for how I evaluate and use AI-generated information even when the system produced the recommendation.

I expect a legitimate school or professional audience to evaluate whether I used GenAI appropriately in important decisions.

## **Decision uncertainty (illustrative)**

In this AI-supported decision, I am uncertain whether the available evidence is sufficient for a justified judgment.

I am unsure how much confidence I should place in the AI-generated recommendation.

I would need additional information before I could be confident in this decision.

I am uncertain whether important contextual information may be missing from the AI-supported analysis.

## **AI literacy – critical evaluation and regulatory use (illustrative)**

I can identify common reasons why a plausible GenAI response may still be inaccurate or misleading.

I can distinguish the fluency of a GenAI response from the strength of the evidence supporting it.

I know how to verify important AI-generated claims using independent or authoritative sources.

I can recognize when a task or type of student information should not be delegated to a general-purpose GenAI system.

I know when uncertainty in an AI-supported judgment warrants consultation, escalation, or an additional human review.

## **Responsible GenAI decision-making (RADM): illustrative behavioral-process indicators**

**Appropriate delegation:**

I use GenAI only for parts of a consequential decision that are suitable for machine assistance.

I keep a task human-only when the educational stakes, privacy requirements, or uncertainty make GenAI assistance inappropriate.

**Verification:**

I verify important AI-generated claims against independent information before acting on them.

I check whether an AI-generated interpretation has omitted relevant learner context.

**Calibrated reliance and accountable human authority:**

I treat GenAI recommendations as inputs to professional judgment rather than as decisions that should be followed automatically.

I modify, reject, or seek another human judgment when AI output is not supported by stronger evidence.

**Traceability:**

I document material GenAI involvement when it contributes to a consequential educational judgment.

I can explain what evidence I checked and why I accepted, modified, or rejected the AI-supported recommendation.

**Escalation and override:**

I seek additional human review when AI-supported evidence remains uncertain or potentially high risk.

I pause or refuse AI-supported action when privacy, fairness, or potential harm cannot be adequately resolved.

**Measurement caution.** RADM is provisionally conceptualized as a higher-order behavioral composite whose dimensions are non-interchangeable. Future validation should compare composite/formative and correlated-factor specifications and should establish criterion validity using scenario choices or behavioral process measures rather than self-report endorsement alone.
